# A One- or Two-Stage Revision of Fungal Prosthetic Joint Infection: A Review of Current Knowledge, Pitfalls and Recommendations

**DOI:** 10.3390/antibiotics14070658

**Published:** 2025-06-30

**Authors:** Hazem Alkhawashki, Joseph Benevenia, Lorenzo Drago, Yazan Kadkoy

**Affiliations:** 1 Advanced Medical Center, Riyadh 12482, Saudi Arabia; 2 Rutgers, New Jersey Medical School Department of Orthopaedics, Newark, NJ 07103, USA; benevejo@njms.rutgers.edu (J.B.); yk399@njms.rutgers.edu (Y.K.); 3 Laboratory of Clinical Medicine with Specialized Areas, IRCCS MultiMedica Hospital, 20138 Milan, Italy; lorenzo.drago@unimi.it; 4 Department of Biomedical Sciences for Health, University of Milan, 20133 Milan, Italy

**Keywords:** fungal, prosthetic infection, one stage, two stages, revision, fungal PJI

## Abstract

Fungal prosthetic joint infection (fPJI) is one of the orthopaedic pathologies where there is no clear evidence, guidelines or algorithm to guide the surgeon in its management. This is in addition to the difficulty with which these infections are diagnosed, isolated and treated. Fungi form notorious biofilms that are difficult to eradicate once formed and that display resistance to antimicrobial agents. These biofilms have been shown to act synergistically with biofilms of bacteria, further adding to medical treatment resistance. We have reviewed the literature for reports that describe the results of different methods in surgically treating fPJI. We found that surgical management with two stages remains the gold standard for treatment of fPJI, as is the case for bacterial PJI (bPJI). We have investigated medical treatment, debridement with implant retention (DAIR) and staged revisions and whether a reasonable recommendation can be made based on the best knowledge and practice available. From the data on bPJI, there exists a role for conservative management of acute PJI with debridement, antibiotics and implant retention (DAIR). While fPJI and bPJI both represent infections, the differences in our ability to detect these infections clinically, culture the pathogens and treat them with proper antimicrobial agents, along with the difference in the reported results of the surgical treatment, make us believe that these two types of infections should not be treated in the same manner. With all this in mind, we reviewed several reports in the literature on fPJI to determine the efficacy of current treatment modalities, including DAIR, which followed current guidelines for PJI. Data show an overall treatment success rate of 64.4% [range 17.4–100%]. Subgroup analysis revealed a success rate of 11.6% [range 0–28.7%] in patients treated with DAIR. There is no doubt that DAIR should not be encouraged as it consistently has a bad record. Although there are not enough studies or numbers of patients to show an evidence-based preference over one- or two-staged revisions, the two-stage revision of fPJI consistently shows better results and should be considered as the gold standard of management in cases of revision fPJI. This should also be coupled with proper expertise, follow-ups and recommended lengths of medical treatment, which should not be less than six months. From the review of these data, we have developed reasonable recommendations for the management of fPJI. These recommendations center on staged surgical debridement along with medical management. Medical treatment should be for at least 6 months under the guidance of an infectious disease team and based on intraoperative cultures. In the case of local antimicrobial treatment reported in the literature, many patients with fPJI were found to have a polymicrobial infection. As a result, it is our recommendation that antifungals as well as antibacterials should be incorporated into the cement spacer mix of these cases. Fungal PJI remains an exceedingly difficult pathology to treat and should be managed by experienced surgeons in a well-equipped institution.

## 1. Introduction

About one hundred, out of thousands, of fungal species cause infection in humans Musser [1,2]. Fungal arthritis has a worldwide distribution with prevalence ranging from 0.4% to 20%, is more prevalent in men and usually presents as oligoarthritis [3]. Fungal prosthetic joint infection (fPJI) ranges between 0.6 and 2% [4,5]. Bone and joint fungal infection most commonly occurs through haematogenous seeding, and more commonly causes osteomyelitis than septic arthritis [1,2,6,7].

The course of fPJI is both insidious and indolent. Clinical symptoms and signs are variable, but pain, swelling and sinus tract are the most common, and are reported in 75–100%, 25–73% and 0–80% of cases, respectively [4,5,6,8,9]. Other variable presentations are low-grade fever, reduced range of motion, warmth, and redness [2].

Fungal PJI is most commonly caused by the Candida albicans and non-albicans species (50–80%), followed by *Aspergillus* [10,11,12,13].

There is poor evidence in the literature regarding the management of fPJI and the main guidelines are based on case reports, case series, reviews, and expert opinions [4,5,14,15,16,17]. Other sources are the consensus of opinions at different infection societies.

We have reviewed the literature and the available knowledge (PubMed, Google Scholar, Orth Evidence, book chapters and infection societies consensus) and developed treatment recommendations based on the best consistent opinion of experts and infection societies, as well as analyses of published articles and results of surgical treatment of fPJI [18,19,20,21,22,23,24,25,26,27,28,29]. The objective of this study was to characterize current surgical treatment modalities for the eradication of the fPJI and their associated patient outcomes.

## 2. Materials and Methods

**Figure 1:**
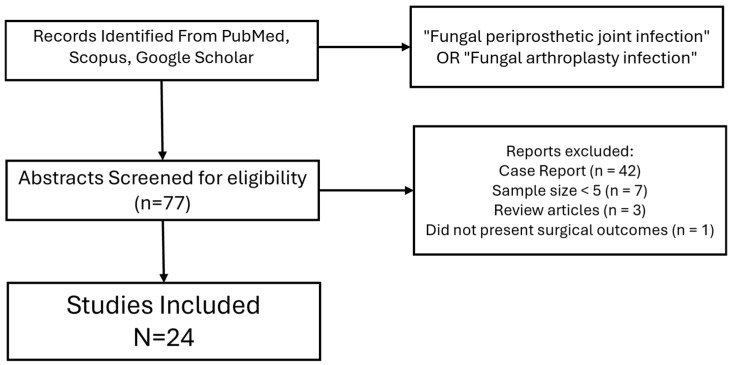
Flow chart of studies. Data on surgical management, treatment success rates, reinfections rates and amputation rates were tabulated for analysis. Due to lack of reporting of individual patient data, we tabulated treatment methods as a percentage of the total number of patients treated. To further characterize current treatment modalities and patient presentation characteristics, patient demographics for age, timing of infection, ESR, CRP, use of antifungal cement and use of antibacterial cement were also tabulated where available. Rates for the DAIR procedure were tabulated separately and were available for comparison of averages for treatment success. ESR = erythrocyte sedimentation rate. CRP = C-reactive protein.

We searched PubMed, Google Scholar, Orth Evidence, book chapters and infection societies consensus and SCOPUS for the following search terms “Fungal periprosthetic joint infection”, “Prosthetic joint infection”, “Fungal infection” and “arthroplasty infection”. We also looked at basic science and medical treatment of fungal infections. For studies involving the surgical management of fungal periprosthetic joint infection (fPJI), we included review articles and only studies written in English. Case reports, technique papers, biomechanical, basic science, and studies with less than 5 patients were excluded. Abstracts were screened for relevance by two reviewers (H.A. and Y.K.). Full papers deemed to fall within the inclusion criteria were then screened to assess eligibility, as shown in Figure 1. Full paper texts deemed to meet the inclusion criteria were discussed between the two reviewers before being included in the analysis. Disputes on study eligibility were discussed between the remaining authors until a unanimous decision was made. Treatment success was defined as no signs of infection at the final timepoint without need for return to OR outside of the intended initial treatment plan.

## 3. Results

A total of 24 studies met our inclusion criteria with 533 patients from 2009 to 2023. The studies represent data from 212 total knee arthroplasties (TKA), 326 total hip arthroplasties (THA), 1 uni-compartmental knee arthroplasty (UKA), and 1 total shoulder arthroplasty (TSA). Surgical management for these patients comprised 90 cases of debridement antibiotics and implant retention (DAIR), 46 one-stage revisions, 329 two-stage revisions and 30 three-stage revisions. The average number of patients per study was 22 ± 15. The average patient age for all studies was 65.4 ± 12.7 years with an average follow up of 42.8 ± 20.4 months. The average CRP provided was 7.47 and the average ESR was 53.1 (Table 1).

The most utilized surgical management method was the two-stage revision within studies with an average of 60%. Overall treatment success rate was 64.4% (range 17–100%) across all studies. On average, 40% of all patients were found to have bacterial coinfection (Table 2). The average rates of amputation are 8%. In total, 14 of the 24 studies (58.3%) described using antifungal cement, namely amphotericin B. Eleven studies describe using antibacterial cement, either in conjunction with or antifungal cement or alone. ‘-’ Denotes value not available in paper.

The average rate of recurrent or persistent infection across all studies was 32%. At final follow-up, an average of 12% of patients had arthrodesis or resection arthroplasty. From the rates reported, an average of 8% of patients treated for fPJI underwent amputation. Overall, for studies where there was recurrent or persistent infection requiring return to OR, the success rate of additional debridement was 34.2%. At the end of all studies, 7.6% of patients remained on suppressive antimicrobial treatment (Table 3). ‘-’ Denotes value not available in paper.

Subgroup analysis for patients treated with DAIR was conducted. Ninety patients were initially treated with DAIR. Of those patients, the average treatment success was 12% (range 0–16%) between studies. Following initial treatment failure, most patients were treated with a two-stage revision. Due to the presentation of these data, a salvage rate for DAIR could not be calculated. Overall, for studies where there was recurrent or persistent infection requiring return to OR, the success rate of additional debridement was 34.2%. At the end of all studies 7.6% of patients remained on suppressive antimicrobial treatment. ‘-’ Denotes value not available in paper.

## 4. Discussion and Literature Review

Prosthetic joint infection (PJI), in general, is a serious complication of joint arthroplasty. It is commonly caused by a variety of microorganisms that include both bacteria and fungi, especially the latter in immunocompromised patients. The incidence of PJI is 1–2%. Fungal PJI is commonly caused by *Candida albicans*.

The most important result of our review is that DAIR consistently gave bad results and a high reoperation rate. Although there was no statistical difference between one- and two-stage revision of fPJI, we recommend two stages as the gold standard of fPJI.

**Figure 2:**
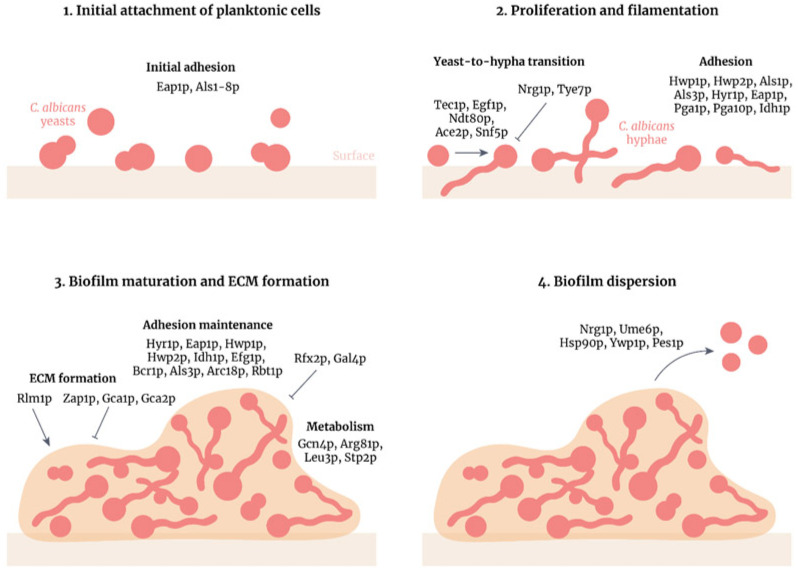
Stages of *Candida albicans* biofilm formation and development. *Candida albicans* biofilm formation is a multifactorial process that consists of the following four main stages. (**1**) Initial attachment of planktonic cells: *C. albicans* yeasts attach to a surface (e.g., epithelia, biomaterials or cellular aggregates) through adhesins. (**2**) Proliferation and filamentation: yeasts transition to hyphae and this process is regulated by many transcription factors. (**3**) Biofilm maturation and extracellular matrix formation: the matrix forms around the *C. albicans* cells, positively regulated by the TF Rlm1p, providing structural support and protection against antifungals and the host immune system. Adhesion is maintained and amino acid metabolism is increased in the biofilm. (**4**) Biofilm dispersion: yeast cells disperse from the biofilm to form colonies in other parts of the body. These cells differ from initial planktonic cells as they are more virulent and more likely to form biofilms. Reprinted from open access reference [38].

A major factor of the virulence of *Candida albicans* is its ability to form biofilms, attaching to biotic and abiotic substrates and forming on synthetic polymers as prosthetic plastics, which are hard to eradicate and are resistant to conventional antifungal treatments [8,38]. *Candida albicans* produces larger and more complex biofilms than other *Candida* species [8]; hence, it is recommended to remove the device affected. Biofilm formation includes four stages, as shown in Figure 2 [38]. Once formed, the biofilm is highly tolerant of antifungal therapy and can serve as a reservoir for recurrent infection.

It is a common occurrence to have a concomitant bacterial infection among cases of fPJI, ranging between 16 and 66%, and most commonly involve *Staphylococcus* species followed by *streptococcus* spp. [4,7,12,39]. The high number of concomitant bacterial infection cases may be explained by the interactions of *C. albicans* with other microorganisms, which can occur via co-aggregation and co-adhesion. *C. albicans* adhesins facilitate interaction with bacterial species such as *Streptococcus gordonii* and *Staphylococcus aureus* [38].

There are several risk factors reported in the literature. Fungal PJI occurs most commonly in immune-compromised patients, those with previous surgeries and revision cases of joint replacement. Riaz et al. reported that the two independent risk factors associated with increased odds of fPJI are antimicrobial therapy within 3 months before PJI diagnosis and prolonged wound drainage exceeding 5 days [4,13]. Other risk factors are chronic disease as diabetes, previous PJI, immunosuppression conditions (chemotherapy, cancer patients, HIV, those on corticosteroids, and organ transplant patients), those with indwelling catheters, those undergoing parenteral nutrition and illicit IV drug users [4,7,10,40,41].

**Figure 3:**
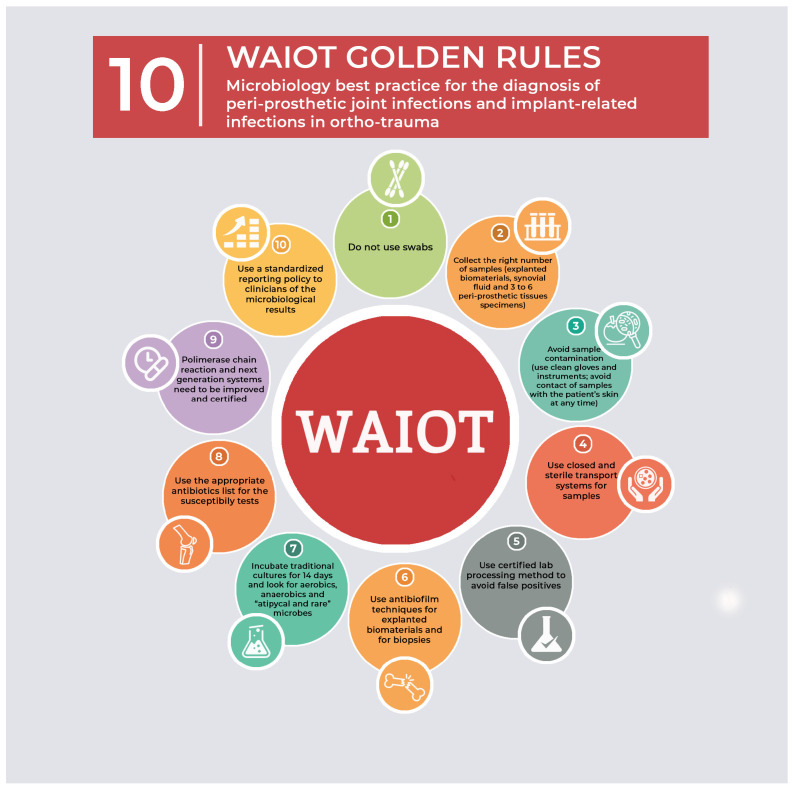
Microbiology best practice for the diagnosis of peri-prosthetic joint infections and implant-related infections in ortho-trauma patients, including the 10 WAIOT golden rules. Reprinted with permission from publisher. Open access reference [42].

To diagnose fPJI, the surgeon should have a high suspension index, especially in high-risk patients. It is not routine to perform all diagnostic tests, except in the most uncertain cases. We recommend following the WAIOT 10 golden rules of sampling for the diagnosis of PJI, as shown in Figure 3 [2,42].

If fPJI is suspected, a traditional X-ray is carried out with leukocyte count, blood culture and serum inflammatory markers (ESR, CRP and D-dimer). The leukocyte count is often normal [5,32,36]. The ESR, CRP and D-dimer markers may be normal or slightly elevated [4,13,43,44]. Reports are often inconsistent, with CRP values between 4 and 31 mg/dL [5,32,36] and ESR values in the normal or low range, below 60 mm/h [5,8,36].

Diagnostic arthrocentesis is performed, unless there is a sinus tract or exposed metal, and surgical debridement is a must [7,16,45]. Aspirate is analysed for low sugar, high protein, and cell count (more than 3000), although they may be of limited value [46]. The aspirate is sent for both bacterial and fungal cultures and the latter takes up to 4 weeks or longer [46]. Intraoperative samples are taken in accordance with the WAIOT 10 rules and sent for cultures and histopathology [2,42].

More sophisticated serology and molecular tests are not readily available, and their clinical use is still under investigation and includes organism-specific antigens, serum beta-glucan, enzyme immunoassays, DNA-based tests and mass spectrometry [11,47]. In some culture-negative cases, uncultivable organisms should be considered, and other identification techniques are performed, such as polymerase chain reaction (PCR) and next generation sequencing (NGS) [4,48].

Despite the WAIOT microbiological rules, challenges related to the identification of fungi and bacteria remain, which often lead to a lack of a standardized approach across clinical settings. In the case of microbiological identification, the diagnostic process generally relies on a combination of culture, molecular methods, and biochemical tests. However, the variability in techniques, interpretation, and availability of resources often leads to inconsistencies in the results. The growing field of molecular diagnostics, including PCR, DNA sequencing, and next-generation sequencing (NGS), has brought significant improvements in the accuracy and speed of pathogen identification. Despite these advances, the integration of these methods into routine clinical practice remains debatable and is often constrained by factors such as cost, availability of skilled personnel, and standardization across laboratories.

In the case of fungi, species identification based on morphological characteristics is often unreliable, especially for closely related species. For example, the identification of *Candida* spp. and *Aspergillus* spp. can be difficult without molecular methods, and even these methods can suffer from lack of standardization [49]. Molecular tools, such as ribosomal RNA sequencing, have been proposed as more accurate alternatives to traditional approaches, but their implementation has not been universally standardized. The World Health Organization (WHO) and the European Society of Clinical Microbiology and Infectious Diseases (ESCMID) have made strides in addressing these issues by publishing guidelines for the diagnosis and management of fungal infections, but widespread adherence remains limited [15].

In the realm of histopathology, confirmatory diagnosis often relies on the detection of pathogens in tissue samples, either through direct microscopy or tissue culture. However, histopathological criteria can also lack standardization, with differences in interpretation, staining techniques, and specimen handling potentially leading to variations in diagnostic outcomes. For example, fungal organisms can often be mistaken for other tissue elements, particularly in immunocompromised patients [15]. Standardized criteria for evaluating histopathological slides would improve diagnostic accuracy and reduce inter-observer variability. Several initiatives, such as the development of the International Society for Human and Animal Mycology (ISHAM) guidelines, are working toward harmonizing such criteria, but progress is still required in terms of global implementation.

Furthermore, the suggestion to store microbiological and histopathological data in a national database is highly relevant. As global antimicrobial resistance (AMR) continues to rise, standardized reporting and database systems can play a pivotal role in tracking outbreaks, identifying resistance patterns, and guiding targeted treatments. The lack of a central repository for microbiological and histopathological data often results in fragmented information, limiting the ability to assess trends and respond effectively to emerging pathogens. Efforts such as the Global Antimicrobial Resistance and Use Surveillance System (GLASS), launched by the WHO, demonstrate the utility of centralized data collection in combating infectious diseases [50]. A similar system for fungal and bacterial infections could potentially improve diagnostic accuracy, guide therapeutic decisions, and inform national and international public health policies.

In conclusion, there is emphasis on the importance of standardization in microbiological and histopathological criteria for diagnosing fungal infections. Establishing more uniform diagnostic protocols, along with creating a national database for storing and analyzing these data, would significantly enhance healthcare delivery and disease surveillance, ultimately benefiting the healthcare system.

The literature lacks both evidence and clear algorithm regarding the best treatment approach in treating fPJI, but there has been preference toward certain surgical approaches and recommendations regarding both medical and surgical managements [4,5,7,10,51].

Regarding medical treatment, the Infectious Disease Society of America (IDSA) guidelines for the duration of treatment with antifungal agents in the treatment of joint arthritis are 6 to 12 months [15].

The European Society for Clinical Microbiology and Infectious Disease recommends, after removal of the implant and proper debridement, to medically treat patient for 14 days with parenteral antifungals, followed by 4–6 weeks of oral agents [4,46,52]. If performing a two-stage revision, the International Consensus Meeting (ICM) recommends a minimum period of 6 weeks of antifungal treatment after the removal of a prosthesis [4,14]. Ueng et al. [27] in his meta-analysis identified a better eradication of infection with prolonged systematic therapy for 3–6 months.

Following surgical debridement, reports have had different approaches in dealing with fungal PJI. The numbers reported are too small to draw firm conclusions, although recent meta-analyses and systematic reviews have shed more light on the best surgical approach in dealing with fPJI. The options at hand are DAIR (Debridement, antibiotics and implant retention), one-stage revision arthroplasty (single-SRA), two-stage revision arthroplasty (two-SRA), three-stage revision arthroplasty (three-SRA), resection arthroplasty, arthrodesis, and amputation [2,4,5,7,10,12,22,39].

It must be noted that amputation, arthrodesis, and resection arthroplasty (RA) may highly diminish the quality of life of the patients and the reported success rate of RA and arthrodesis from small series and heterogeneous reports is 80% and 67%, respectively [7,41], and that of amputation is 66% only [23]. Therefore, they should be sought as a salvage procedure in the highest resistance cases or those with repeated uncontrollable infection despite multiple procedures and long antifungal treatment. The reported surgical treatment methods come from small case series, case reports and systematic reviews, and are heterogeneous [4,5,7,10,15,30,41,51,53].

Debridement, antibiotic, and implant retention (DAIR) have been used in small number of cases and mainly in cases reported within 4 weeks of the primary procedure and often resulting in persistent infection and only 20–30% success rate [4,5,36,39]. For bacterial PJI, the consensus is that chronic infections should never be treated with DAIR, and the same has been suggested for fPJI, unless revision surgery is contraindicated or refused by the patient after adequate information [16,39,54].

In a recent systematic review, Sambri et al. [4] and Koutserimpas et al. [7] reported a predominance favoring Two-SRA, (64.2%) and (54%), respectively. Other authors also reported a preference of Two-SRA among surgeons [8,10,14,23,39,53]. A success rate of over 92% has been reported for Two-SRA [7,35,54], although there is wide variability of success rate. Anagnostakos et al. [35] reported a 100% success in a small series of 7 patients. Haleem et al. reported infection eradication up to above 90% [54]. Kuiper et al. [39] reported an 84.8% success rate, and Phelan et al. [55] reported 80%. On the other hand, Azzam et al. [8] reported a 47.4% success rate only. It is notable that the success rate diminishes in the case of bacterial co-infection [7].

The optimal time interval for reimplantation is unknown. A minimum of six weeks is usually recommended [10,39], although this is extended to 3–6 months in revision and re-revision cases [7]. In all cases reimplantation is performed when the clinical picture and blood markers have come to normal picture [39]. There is no conclusive evidence to support the use of an antimicrobial holiday period prior to reimplantation in case of fungal PJI treated with staged revision [12].

The Single-SRA has been used and reported with discordant results. A success rate of 90% has been reported by Klatte et al. [32] in a small series of 10 patients. On the other hand, Ji et al. [30] reported a recurrence rate of 36%. Others have reported a 75% success rate [7]. Multiple studies have demonstrated failed treatment when using DAIR in this patient population, especially in the setting of concomitant bacterial infection [25,27,28,34,36]. A Single-SRA might be considered in patients with unfit medical situations or those who may not tolerate multiple procedures, along with prolonged anti-fungal therapy. Some papers on the one stage treatment of fPJI have shown promising results which are comparable to those from two stage revision [30,32] However, these results have not been repeated on a large scale. The authors of single stage revision stress the importance of having predicable cultures and sensitivities at the time of reimplantation.

While two-SRA appears as the most adopted surgical approach, there are no comparative studies showing its superiority over single-SRA.

### Limitations of This Study

The limitations of the review articles include author bias, incomplete coverage, single timeframe and oversimplification of complex issues. In addition, they are subject to systematic and random errors, arising from (a) the selection of studies included in the analysis, (b) the validity of studies selected for the analysis, (c) publication bias, (d) small sample sizes, and (e) the heterogeneity of methods used in the studies reviewed. Although there is an increasing number of fungal PJI cases, the research on this topic is limited and the authors are not aware of published, prospective, randomized trials on the treatment of this condition. There is also a lack of studies limited to a specific fungal species. This lack of information limits healthcare providers and specific treatment recommendations. There are also several limitations of this study. First, it is based upon review articles from retrospective studies, different institutions and treatment regimens, differing in their use of antibiotics in oral, parenteral, and cement. The individual patient data that were analyzed are limited and not uniform. Overall, there was an overwhelming number of surgeries described as two-stage (329) compared to one-stage treatments (46) and DAIR (90). These data are overwhelmingly weighted towards treatment using two-stage methods.

## 5. Conclusions

According to the author’s review of the literature, fungal PJI is a difficult condition to treat. There is a paucity of high-level data to report statistical significance. If treatment success is based on no re-operations, the highest salvage rate reported is the use of the so-called two stage method and the lowest by the DAIR procedure. Overall, specific treatment for each patient must be decided by the surgical team only after open discussion with the patient and based on the general and local conditions, the clinical history and the expectations of the patient.

## Figures and Tables

**Table 1:** Summary of patient characteristics in included studies.

Study	Year	*n*	Average Age	Follow Up (Months)	Average ESR (mm/h)	Average CRP (mg/L)	Coinfection
Herndon et al. [18]	2023	41	64	12	-	-	35
Yang et al. [19]	2023	41	77.6	38.9	60.5	7.9	13
Zhang at al. [20]	2022	11	59	34.5	66.1	2.04	0
McCulloch et al. [21]	2022	69	68	34	-	-	63
Baecker et al. [22]	2021	18	72	35	-	-	6
Enz et al. [23]	2021	18	70	-	-	-	14
Karczewski et al. [24]	2021	29	70.98	33.03	-	5.17	22
Saconi et al. [5]	2020	11	65	42.5	34.8	31.2	6
Theil et al. [12]	2019	26	72	27	-	-	13
Sidhu et al. [25]	2019	22	64	46.2	47.6	6.2	22
Gao et al. [26]	2018	18	61	65	57.5	4.4	9
Kou et al. [27]	2018	29	70.7	86.4	59.2	6.6	15
Escolà-Vergé et al. [28]	2018	35	75	16.5	66	3.8	11
Brown et al. [29]	2017	31	9	48	41.0	5.3	4
Ji et al. [30]	2017	11	66	12	-	-	6
Kim et al. [31]	2017	9	76	66	56	2.25	3
Klatte et al. [32]	2014	10	68	96	-	22	1
Geng et al. [33]	2014	8	60	55	68	4.7	4
Wang et al. [9]	2014	5	67	42	40	2.3	0
Ueng et al. [34]	2012	16	62	41	-	-	8
Anagnostakos et al. [35]	2012	7	68	28	-	-	0
Hwang et al. [36]	2011	30	69	52	39	4.3	6
Dutronc, et al. [37]	2010	7	72	30	-	9.8	0
Azzam et al. [8]	2009	31	64	45	54	1.75	5

**Table 2:** Summary of surgical management by study.

Study	Year	*n*	THA	TKA	DAIR	One-Stage	Two-Stage	Three-Stage	Coinfection	Treatment Success
Herndon et al. [18]	2023	41	19	22	14%	0%	27%	0%	35	49
Yang et al. [19]	2023	41	0	41	24%	0%	100%	0%	13	63
Zhang at al. [20]	2022	11	1	9	0%	9%	91%	0%	0	100
McCulloch et al. [21]	2022	69	22	47	23%	7%	70%	0%	63	17
Baecker et al. [22]	2021	18	11	7	0%	0%	0%	100%	6	89
Enz et al. [23]	2021	18	14	4	5%	0%	39%	0%	14	50
Karczewski et al. [24]	2021	29	14	15	7%	7%	28%	41%	22	62
Saconi et al. [5]	2020	11	6	5	9%	36%	9%	0%	6	73
Theil et al. [12]	2019	26	18	8	0%	8%	92%	0%	13	38
Sidhu et al. [25]	2019	22	8	14	32%	0%	68%	0%	22	41
Gao et al. [26]	2018	18	5	13	0%	0%	100%	0%	9	72
Kou et al. [27]	2018	29	14	15	24%	10%	65%	0%	15	41
Escolà-Vergé et al. [28]	2018	35	26	16	43%	20%	22%	0%	11	49
Brown et al. [29]	2017	31	13	18	13%	3%	67%	0%	4	61
Ji et al. [30]	2017	11	4	7	0%	100%	0%	0%	6	64
Kim et al. [31]	2017	9	0	9	78%	0%	100%	0%	3	89
Klatte et al. [32]	2014	10	6	4	0%	100%	0%	0%	1	90
Geng et al. [33]	2014	8	4	4	0%	0%	100%	0%	4	75
Wang et al. [9]	2014	5	0	5	0%	0%	100%	0%	0	100
Ueng et al. [34]	2012	16	7	9	0%	0%	56%	0%	8	50
Anagnostakos et al. [35]	2012	7	4	3	0%	0%	100%	0%	0	100
Hwang et al. [36]	2011	30	0	30	13%	0%	87%	0%	6	93
Dutronc, et al. [37]	2010	7	3	4	14%	0%	71%	0%	0	43
Azzam et al. [8]	2009	31	14	17	23%	0%	94%	0%	5	35

**Table 3:** Summary of treatment outcomes.

Study	Year	Reinfection	Resection/Arthrodesis	Failed Reimplantation	Amputation	Initial Failure	Salvage	Suppression
Herndon et al. [18]	2023	51.2	17.1	22.0	4.9	0.0		17.1
Yang et al. [19]	2023	36.6	34.1	29.3	0.0	36.6	0.0	31.7
Zhang at al. [20]	2022	0.0	0.0	27.3	0.0	0.0		0.0
McCulloch et al. [21]	2022	49.3	10.1	20.3	11.6	49.3	47.1	42.0
Baecker et al. [22]	2021	11.1	22.2	0.0	0.0	11.1	50.0	0.0
Enz et al. [23]	2021	22.2	5.6	0.0	33.3	22.2	0.0	16.7
Karczewski et al. [24]	2021	27.6	27.6	27.6	0.0	27.6	0.0	0.0
Saconi et al. [5]	2020	9.1	0.0	0.0	9.1	9.1	0.0	9.1
Theil et al. [12]	2019	23.1	7.7	7.7	3.8	61.5	18.8	0.0
Sidhu et al. [25]	2019	59.1	4.5	0.0	4.5	36.4	0.0	9.1
Gao et al. [26]	2018	27.8	11.1	11.1	5.6	22.2	75.0	0.0
Kou et al. [27]	2018	58.6	37.9	41.4	3.4	69.0	5.0	0.0
Escolà-Vergé et al. [28]	2018	51.4	5.7	5.7	2.9	51.4	22.2	14.3
Brown et al. [29]	2017	38.7	16.1	6.5	3.2	38.7	33.3	19.4
Ji et al. [30]	2017	27.3	0.0	0.0	9.1	18.2	100.0	0.0
Kim et al. [31]	2017	88.9	0.0	0.0	11.1	77.8	100.0	0.0
Klatte et al. [32]	2014	10.0	0.0	0.0	10.0	10.0	100.0	0.0
Geng et al. [33]	2014	25.0	12.5	0.0	12.5	25.0	50.0	0.0
Wang et al. [9]	2014	0.0	0.0	0.0	20.0	0.0	-	0.0
Ueng et al. [34]	2012	50.0	43.8	43.8	6.3	50.0	0.0	0.0
Anagnostakos et al. [35]	2012	0.0	0.0	28.6	14.3	0.0	-	14.3
Hwang et al. [36]	2011	20.0	3.3	0.0	3.3	6.7	50.0	0.0
Dutronc, et al. [37]	2010	42.9	0.0	0.0	14.3	42.9	0.0	0.0
Azzam et al. [8]	2009	32.3	19.4	0.0	3.2	38.7	33.3	9.7

## Data Availability

No new data were created or analyzed in this study.
